# Transcriptomic changes in porcine articular cartilage one year following disruption of the anterior cruciate ligament

**DOI:** 10.1371/journal.pone.0284777

**Published:** 2023-05-03

**Authors:** Jonah I. Donnenfield, Naga Padmini Karamchedu, Benedikt L. Proffen, Janine Molino, Braden C. Fleming, Martha M. Murray

**Affiliations:** 1 Division of Sports Medicine, Department of Orthopaedic Surgery, Boston Children’s Hospital, Harvard Medical School, Boston, MA, United States of America; 2 Department of Orthopaedics, Warren Alpert Medical School of Brown University/Rhode Island Hospital, Providence, RI, United States of America; University of Vermont College of Medicine, UNITED STATES

## Abstract

To determine the transcriptomic changes seen in early- to mid-stage posttraumatic osteoarthritis (PTOA) development, 72 Yucatan minipigs underwent transection of the anterior cruciate ligament. Subjects were randomized to no further intervention, ligament reconstruction, or ligament repair, followed by articular cartilage harvesting and RNA-sequencing at three different postoperative timepoints (1, 4, and 52 weeks). Six additional subjects received no ligament transection and provided cartilage tissue to serve as controls. Differential gene expression analysis between post-transection cartilage and healthy cartilage revealed an initial increase in transcriptomic differences at 1 and 4 weeks followed by a stark reduction in transcriptomic differences at 52 weeks. This analysis also showed how different treatments genetically modulate the course of PTOA following ligament disruption. Specific genes (e.g., *MMP1*, *POSTN*, *IGF1*, *PTGFR*, *HK1*) were identified as being upregulated in the cartilage of injured subjects across all timepoints regardless of treatment. At the 52-week timepoint, 4 genes (e.g., *A4GALT*, *EFS*, *NPTXR*, *ABCA3*) that—as far as we know—have yet to be associated with PTOA were identified as being concordantly differentially expressed across all treatment groups when compared to controls. Functional pathway analysis of injured subject cartilage compared to control cartilage revealed overarching patterns of cellular proliferation at 1 week, angiogenesis, ECM interaction, focal adhesion, and cellular migration at 4 weeks, and calcium signaling, immune system activation, GABA signaling, and HIF-1 signaling at 52 weeks.

## Introduction

Recent studies estimate there may be as many as 250,000 ACL tears per year in the United States [1, 2]. In addition to the multibillion-dollar toll these injuries put on the healthcare system, they can devastate the psychological and physical well-being of young athletes who comprise the majority of those injured [1, 3, 4]. In addition, the gold standard treatment for this condition, ACL reconstruction, has shown little-to-no benefit in terms of staving off one of the most common complications of ACL injury: posttraumatic osteoarthritis (PTOA) [4].

PTOA, which accounts for roughly 12% of all symptomatic osteoarthritis cases, develops within 15 years of ACL injury in about half of individuals [4, 5]. This, combined with the skew towards younger incidence, means the duration of disease burden per individual is generally longer than with idiopathic osteoarthritis. Despite the demographically wide-reaching and enduring aspects of PTOA, there remains no FDA-approved, disease-modifying treatment for it or even the more general form of osteoarthritis. In part, this is due to an incomplete understanding of how osteoarthritis develops.

In the past two decades, scientific advances in RNA sequencing (RNA-Seq) have provided a tool to study osteoarthritis. Gene expression of cartilage from humans undergoing total knee arthroplasty has been characterized in end-stage osteoarthritis [6–9], and preclinical studies have described the early gene expression changes upon induction of PTOA in animals (e.g., mice [10–12], dogs [13, 14], horses [15, 16], pigs [17]). However, none of these studies sequenced the cartilage beyond 16 weeks. Thus, even with these new tools, we still need mid-stage disease characterization.

To address this gap, we analyzed the transcriptomes of 90 cartilage samples from 78 Yucatan minipigs with healthy cartilage and PTOA cartilage at three different timepoints (1, 4, and 52 weeks) in disease development. This porcine model, and these animal cohorts in particular, has previously been shown to develop significant macroscopic cartilage damage at six months, with worsening at 12 months, even when treated with different methods of ligament stabilization [18, 19]. The current study applied updated differential gene expression analysis algorithms to determine the changes seen in early- to mid-stage PTOA development to help address the current knowledge gap in our field.

## Methods

### Study design

Seventy-eight adolescent Yucatan minipigs (Sinclair BioResources, Columbia, MO) were included in this study and came from two cohorts that have previously been reported on [17, 19]. Approval from the Brown University Institutional Animal Care and Use Committee was obtained prior to these studies (Protocol number: 1511000175), which were designed following the ARRIVE guidelines [20]. In the first (short-term) cohort, 36 Yucatan minipigs were allocated to undergo unilateral ACL transection (N = 36) followed by no further treatment (ACLT) or surgical repair (REPAIR) or reconstruction (RECON) of the ligament (N = 6 per group at 1 and 4 weeks). Six additional minipigs were allocated not to receive surgery (controls, N = 6). In the second (long-term) cohort, 36 Yucatan minipigs were randomized to ACLT (N = 12) or surgical treatment with RECON or REPAIR (N = 12 per group), followed by euthanasia and outcome assessment at 52 weeks (N = 36) after surgery. To establish controls for the long-term cohort, computerized randomization stratified by sex, surgical group, and knee laterality selected 4 contralateral knees from each surgical group to establish a total of 12 control samples to which surgical joints at 52 weeks would be compared against. Justification for the Yucatan minipig model, details for the IACUC-approved surgical procedures, animal husbandry, pain management have been reported and can be found in S1 Appendix along with the IACUC approval letter [17, 19]. After surgical operation, investigators were blinded to animal group assignments for all outcome assessments in both short-term and long-term studies.

### Macroscopic cartilage evaluation and extraction

Immediately after euthanasia, knee joints were assessed for macroscopic damage to the articular cartilage surface by Osteoarthritis Research Society International (OARSI) guidelines for sheep and goat [21]. Cartilage assessment was performed by two orthopedics-trained physicians who arrived at a verbal consensus for each subject. Thus, there was no quantitative inter-observer analysis, but the same Yucatan minipig PTOA model was previously used by the group conducting the current study, and it was determined in Fleming et al. 2015 that the macroscopic damage score was reliable between independent examiners (intraclass correlation coefficient = 0.96) [22]. Damage to six articular surfaces, the medial femoral condyle, medial tibial plateau, lateral femoral condyle, lateral tibial plateau, femoral trochlea, and patella, were scored from 0 (normal) to 4 (large erosions down to subchondral bone). These scores were summed to create a macroscopic score sum that could range from 0 to 24. After cartilage assessment, osteochondral samples were extracted from the medial femoral condyle—with RNA isolation samples coming from the surface posterior to the frontal plane in the center of the medial femoral condyle. 4–8 5mm osteochondral biopsies were taken from each animal, and these samples were subsequently rinsed with water and separated from the attached bone. They were then flash frozen in liquid nitrogen and placed in a -80°C freezer until homogenization and RNA isolation.

### Cartilage RNA-seq

Sample processing methods, from tissue extraction to RNA sequencing, for the 1- and 4-week samples can be found in Sieker et al. 2018 [17], while the 52-week samples were extracted de novo for this study. In brief, all samples were homogenized in 2 ml tubes (MP Biomedical) containing 500 μl of frozen TRIzol (Life Technologies) using a sterile drill bit while tubes were submerged in liquid nitrogen [17]. After one round of drilling, tubes received an additional 500 μl of liquid TRIzol, were flash-frozen in liquid nitrogen, and again homogenized using the drill bit. Total RNA was extracted using phenol-chloroform and purified using PureLink RNA Mini Kit (Life Technologies). Samples were then checked for purity with NanoDrop (Thermo Scientific) and checked for integrity using a combination of Agilent Tapestation High Sensitivity RNA Screen Tape and Agilent Tapestation RNA Screen Tape. For the 52-week samples, mean 260/280 absorbance ratios were 1.71 and 1.81 for controls and surgical groups, respectively. Mean 260/230 ratios were 1.22 and 1.46, and mean RIN integrity numbers (RINs) were 6.1 and 5.6, respectively (Table 1). All 52-week samples were then library-prepped using KAPA mRNA HyperPrep with RiboErase (Roche) and subsequently pooled and sequenced on a NovaSeq 6000 S2 Flow Cell with 100-bp paired-end reads (Biopolymers Facility, Harvard Medical School, Boston, MA). Newly generated fastq files for the 52-week samples, along with the legacy, short-term fastq files, were piped into FastQC version 0.11.9 to create individual sample reports that were then compiled using MultiQC version 1.12 [23, 24]. Summary statistics and histograms of mean quality values across each base position of the read were generated (S2 Appendix). All samples were contained within a Phred score range of 31.80 to 39.36. Using Salmon version 1.8.0, reads were then quasi-mapped, and transcript quantification files were generated [25]. For 1-, 4-, and 52-week surgical samples, the mean number (and %) of uniquely mapped reads was 14.2 million (93.3%), 10.1 million (81.7%), and 30.8 million (78.2%), respectively. The mean number of uniquely mapped reads for 1- and 4-week controls was 11.6 million (81.8%); for 52-week controls, it was 29.7 million (78.2%). Mapping employed the Sscrofa11.1 porcine genome, which was assembled by The Swine Genome Sequencing Consortium (SGSC) and hosted by Ensembl (http://ftp.ensembl.org/pub/current_embl/sus_scrofa/) [26]. STAR version 2.7.3a and Qualimap version 2.2.1 were also used to align and document alignment statistics, respectively, and the summary statistics from these programs can be found in the data availability repository. Differential gene expression analysis and functional pathway analysis were performed as detailed below.

**Table 1:** RNA quality and sequencing characteristics.

	*52W CON*	*52W Pooled*	*52W Pooled/CON*
	*Mean (Range)*	*Mean (Range)*	*P* ^*a*^
***Concentration (ng/μl)***	17.2 (1.59, 33.8)	27.3 (7.53, 53.2)	0.029
***260/280 Absorbance***	1.71 (1.46, 1.91)	1.81 (1.60, 1.94)	0.044
***260/230 Absorbance***	1.22 (0.35, 1.76)	1.46 (0.94, 2.02)	0.097
***RIN***	6.1 (4.8, 7.3)	5.6 (4.7, 7.0)	0.055
***Uniquely mapped reads in millions***	29.7 (25.3, 35.2)	30.8 (15.9, 46.6)	0.455
***Uniquely mapped reads %***	78.2 (69.0, 83.6)	78.2 (70.6, 86.2)	0.973

^a^ Independent two sample t-tests.

RNA quality and sequencing characteristics for 52-week (52W) pooled surgical subjects (Pooled) and control subjects (CON). Ideal 260/280 and 260/230 absorbance ratios are approximately 2.0. RNA integrity numbers (RIN) above 5 are acceptable for samples having undergone rRNA-depleted library prep.

### Statistical analysis

Demographic and RNA quality characteristics for within-study comparisons (i.e., comparisons between 1-week and 4-week surgical subjects or between 52-week surgical subjects and 52-week controls) were analyzed with independent two sample t-tests. Cartilage score comparisons between short-term controls and long-term controls were analyzed with independent two sample t-tests. Four-surface scores were used for these comparisons as the short-term controls did not undergo assessment of the patella and trochlea like the long-term contralateral controls. One-way ANOVA tests were used to assess demographic differences across all subject groups (i.e., comparisons among 1-week, 4-week, and 52-week surgical subjects and controls). Randomization tests with 200 permutations were performed on differential gene expression lists to determine if the number of significant genes produced by a comparison was robust to sample label randomization. P-values ≤ 0.05 were considered statistically significant. A Gaussian mixture model, similar to that used by Donnenfield et al. 2022, was employed to soft cluster 52-week surgical subjects into two subpopulations based on macroscopic cartilage damage score sums [27]. Subjects were labeled as having either “less” or “more” damaged cartilage based on which subpopulation they had a greater than 50% chance of belonging to. “Less” damaged subjects were compared to “more” damaged subjects for differential gene expression analysis.

### Differential gene expression analysis

Differential gene expression analysis was performed in R version 4.2.1 using DESeq2 on transcript quantification files produced by Salmon mapping [28, 29]. Gene outliers were handled using the DESeq2 default criteria for zero counts, extreme counts, and low mean normalized counts. P-values were adjusted for multiple testing using the Benjamini-Hochberg false discovery rate (FDR) with a value of < 0.05 as the cutoff for significance.

### Functional pathway analysis

Overrepresentation analysis employed hypergeometric testing on the significantly differentially expressed gene lists and tested for representation of Gene Ontology (GO) terms [30]. These terms included biological processes, molecular functions, and cellular components, and the priority of reporting these terms was assigned in that order (i.e., if the biological process and molecular function terms were present, biological process terms were documented in the main text with molecular function terms and all other GO terms in S3 Appendix in the data availability repository). Because overrepresentation analysis is dependent upon statistically significant differentially expressed genes, it was only performed on comparisons that produced gene lists that were robust to randomization testing. Clusterprofiler was used to create dotplots and category netplots of the GO terms, and Revigo treemaps grouped GO terms by parent terms for high-level visualization [31, 32]. Dotplots were used in cases where many GO terms were present and highlighting significance among them was prioritized. Category netplots were used in cases where fewer GO terms were present, and there was an emphasis on showcasing influential genes. Gene Set Enrichment Analysis (GSEA) was performed to assess Kyoto Encyclopedia of Genes and Genomes (KEGG) pathway enrichment independent of significant gene lists produced by DESeq2 [33, 34]. Signaling Pathway Impact Analysis (SPIA) was also used to evaluate significant KEGG pathways in a way that was more statistically robust than GSEA alone (i.e., SPIA incorporated a combination of hypergeometric testing and permutation testing) [35]. Complete lists of GSEA and SPIA results for all groups comparisons can be found in S4 Appendix in the data availability repository. An adjusted p-value cutoff of < 0.05 was used for all pathway analysis methods to determine significance.

## Results

There were no differences in baseline age, weight, or sex among the 1-week and 4-week surgical subjects or their controls [17]. There were no demographic differences between 52-week surgical subjects or their controls (Table 2). There was also no difference in the macroscopic cartilage scores in the control knees for the short-term and long-term control groups (Table 2).

**Table 2:** Demographic and macroscopic cartilage data.

	*1-4W*	*1W*	*4W*	*52W*	*52W*	*52W*	*All*	*52W CON/*
	*CON*	*Pooled*	*Pooled*	*CON*	*Pooled*	*Pooled/CON*	*Groups*	*1-4W CON*
	*Mean (Range)*	*Mean (Range)*	*Mean (Range)*	*Mean (Range)*	*Mean (Range)*	*P* ^*a*^	*P* ^*b*^	*P* ^*a*^
***Age (mo)***	17.3	17.5	17.2	15.0	15.3	0.590	2.25E-8	
	(16.1, 18.4)	(15.5, 20.3)	(15.3, 18.7)	(13, 17)	(12, 18)			
***Weight (kg)***	53.3	54.1	53.8	50.8	52.0	0.545	0.190	
	(50, 58)	(47, 60)	(47, 60)	(40, 60)	(40, 60)			
***Female as %***	50%	50%	50%	50%	50%	1	1	
***Cartilage damage***								
***MTP***	0.3 ± 0.5			0.6 ± 0.7				0.400
***LTP***	0.0 ± 0.0			0.2 ± 0.4				0.166
***MFC***	0.5 ± 0.8			0.8 ± 1.0				0.581
***LFC***	0.2 ± 0.4			0.2 ± 0.4				1
***4-surface sum***	1.0 ± 1.5			1.7 ± 1.6				0.410

^a^ Independent two sample t-tests.

^b^ One-way single factor ANOVAs.

Baseline demographic data for subjects from both the current and prior studies are shown. Macroscopic cartilage data (mean ± standard deviation) from short-term controls (1-4W CON) and long-term controls (52W CON) are shown for four articular surfaces: medial tibial plateau (MTP), lateral tibial plateau (LTP), medial femoral condyle (MFC), and lateral femoral condyle (LFC).

### Differential gene expression analysis by experimental group

**Fig 1:**
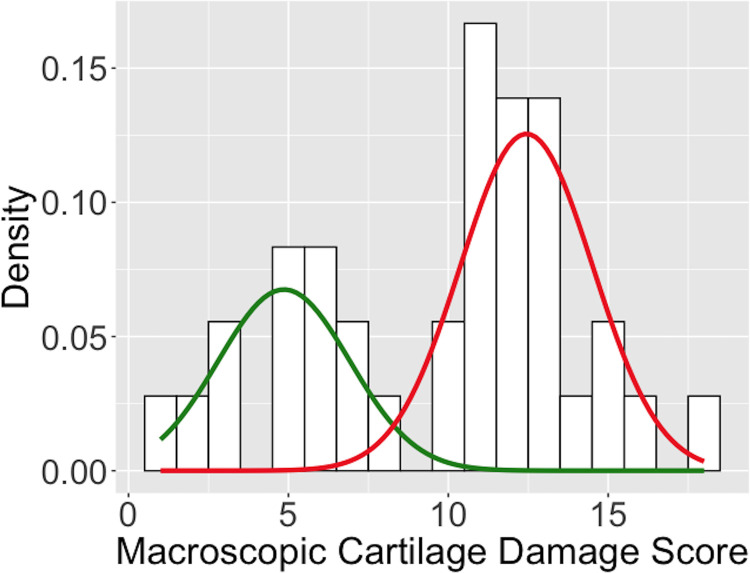
Gaussian mixture model plot. Green and red lines represent the different gaussian distributions for “less” damaged and “more” damaged cartilage groups, respectively. The optimal separation was between macroscopic sum scores of 8 and 9.

Expression differences between surgical groups and controls were more significant than those among surgical groups, where only REPAIR and RECON were significantly different at 4 and 52 weeks (Table 3; exact p-values shown in Table 1 in S5 Appendix). The top differentially expressed genes between surgical groups and controls at each timepoint are shown in Table 4. Based on the gaussian mixture model, 52-week surgical subjects with 6-surface cartilage damage scores ≥ 9 had a greater than 50% probability of belonging to the “more” damage group (N = 23), and surgical subjects with scores < 9 had a greater than 50% probability of belonging to the “less” damaged group (N = 13) (Fig 1). There was no significant difference in gene expression between these groups (Table 3). This lack of expression heterogeneity among surgical groups justified pooling surgical groups for timepoint comparisons.

**Table 3:** Number of differentially expressed transcripts for each group comparison.

		*1-Week*		*4-Week*				*52-Week*	
	ACLT	RECON	REPAIR	ACLT	RECON	REPAIR	ACLT	RECON	REPAIR
***Controls***	2032*	2148*	3203*	3131*	3951*	4237*	65*	273*	164*
***ACLT***		5	17		28	1		1	3
***RECON***			1			37*			47*
								**Cartilage damage ≥ 9**	
							**Cartilage damage ≤ 8** 3		
	***Shared transcripts (top 100)***			***Shared transcripts (top 100)***			***Shared transcripts (top 100)***		
		1281 (68)			1824 (38)			20 (11)	
		2 (2)			0 (0)			0 (0)	
		--			--			--	
	***Unique transcripts***			***Unique transcripts***			***Unique transcripts***		
	0	0	0	3	15	1	24	47	46
		3	15		28	1		1	3
			--			--			--

The number of differentially expressed transcripts for each group comparison (p ≤ 0.05 represented by *). Shared transcripts among comparisons are shown below their respective timepoints with values in parentheses indicating how many transcripts are shared when the transcript list is narrowed to its top 100 most statistically significant transcripts. Unique transcripts (i.e., present in the top 100 most significant transcripts for a given comparison and absent from the top 500 most significant transcripts for similar comparisons) for each group comparison are also shown beneath their respective timepoints.

**Table 4:** Top 20 differentially expressed transcripts.

A	Gene Symbol	Description	ACLT/CON L2FC	RECON/ CON L2FC	REPAIR/ CON L2FC	REPAIR/ CON Adjusted P-value
1	*RRM2*	ribonucleotide reductase regulatory subunit M2	2.92	3.34	3.68	6.16E-27
2	*NCAPH*	non-SMC 11pregulate I complex subunit H	2.61	3.05	3.08	1.17E-23
3	*TYMS*	thymidylate synthetase	2.93	3.26	3.50	1.17E-23
4	*UHRF1*	ubiquitin like with PHD and ring finger domains 1	3.09	3.47	3.83	1.23E-22
5	*CDCA7*	cell division cycle associated 7	3.26	3.72	4.22	2.91E-22
6	LDHA	lactate dehydrogenase A	1.78	1.70	2.07	1.04E-21
7	*SAA3*	serum amyloid A 3	5.55	6.79	8.84	5.84E-21
8	*TPX2*	TPX2 microtubule nucleation factor	2.89	3.16	3.03	1.14E-20
9	*LMNB2*	lamin B2	1.27	1.45	1.60	2.61E-20
10	*CDCA8*	cell division cycle associated 8	3.00	3.32	3.30	4.03E-20
11	*CKS2*	CDC28 protein kinase regulatory subunit 2	2.57	2.66	2.61	9.58E-20
12	*STMN1*	stathmin 1	2.80	3.02	2.88	3.05E-19
13	*ASF1B*	anti-silencing function 1B histone chaperone	3.14	3.64	3.66	6.03E-19
14	*CCNB2*	cyclin B2	3.19	3.51	3.39	8.02E-19
15	*TCF19*	transcription factor 19	3.01	3.07	3.45	3.86E-18
16	*COCH*	cochlin	-3.97	-6.39	-5.97	5.16E-18
17	*ZNF367*	zinc finger protein 367	2.91	3.06	3.47	5.37E-18
18	*NCAPG*	non-SMC 11pregulate I complex subunit G	3.03	3.22	3.21	5.51E-18
19	*CKAP2*	cytoskeleton associated protein 2	3.85	4.16	4.04	9.81E-18
20	*CENPN*	centromere protein N	2.80	2.76	2.90	3.04E-17
**B**						
1	*RGS16*	regulator of G protein signaling 16	2.37	3.27	2.92	1.86E-14
2	*COL6A3*	collagen type VI alpha 3 chain	1.88	2.55	2.11	3.63E-13
3	*SPOCK1*	SPARC (osteonectin), cwcv and kazal like domains proteoglycan 1	3.89	4.41	4.35	4.73E-13
4	*AQP1*	aquaporin 1 (Colton blood group)	4.94	4.74	5.20	4.73E-13
5	*IFNAR2*	interferon alpha and beta receptor subunit 2	1.62	1.55	1.65	6.81E-13
6	*IGF1*	insulin like growth factor 1	4.69	4.28	4.87	7.05E-13
7	*C1QTNF3*	C1q and TNF related 3	3.34	3.87	3.49	7.30E-13
8	*ADAMTS12*	ADAM metallopeptidase with thrombospondin type 1 motif 12	2.99	3.03	3.62	1.10E-12
9	*MMP1*	matrix metallopeptidase 1	7.39	7.29	7.82	2.49E-12
10	*PTGFR*	prostaglandin F receptor	5.48	5.32	5.60	4.39E-12
11	*POSTN*	Periostin	6.76	5.47	5.90	9.94E-12
12	*THY1*	Thy-1 cell surface antigen	3.23	3.09	3.43	1.26E-11
13	*PTGFRN*	prostaglandin F2 receptor inhibitor	1.47	1.55	1.47	1.57E-11
14	Orthologue: *IGHV3*	Immunoglobulin Heavy Chain Variable Region	17.07	19.12	14.79	1.98E-11
15	*BACE2*	beta-secretase 2	2.43	2.30	2.64	3.59E-11
16	*SDK1*	sidekick cell adhesion molecule 1	3.25	2.94	3.57	3.10E-10
17	*GRIP1*	glutamate receptor interacting protein 1	2.25	2.30	2.50	3.22E-10
18	*MATN2*	matrilin 2	2.00	1.81	2.09	1.90E-09
19	novel gene	NA	-27.66	-28.60	-27.68	1.94E-09
20	*CAPZB*	capping actin protein of muscle Z-line subunit beta	0.91	1.06	1.06	2.34E-09
**C**						
1	*HTR7*	5-hydroxytryptamine receptor 7	0.98	1.15	1.35	4.58E-05
2	*TRAK1*	trafficking kinesin protein 1	0.24	0.54	0.45	3.68E-04
3	*HS6ST1*	heparan sulfate 6-O-sulfotransferase 1	0.38	0.46	0.63	4.29E-04
4	*LAMA4*	laminin subunit alpha 4	0.58	0.80	0.56	1.77E-03
5	*A4GALT*	alpha 1,4-galactosyltransferase	0.46	1.11	0.83	4.76E-03
6	*PDE4A*	phosphodiesterase 4A	0.34	0.53	0.34	9.82E-03
7	*GPNMB*	glycoprotein nmb	2.49	2.69	1.62	1.01E-02
8	*EFS*	embryonal Fyn-associated substrate	0.39	0.35	0.36	1.03E-02
9	*NPTXR*	neuronal pentraxin receptor	-0.32	-0.54	-0.42	1.43E-02
10	*ABCA3*	ATP binding cassette subfamily A member 3	0.56	0.72	0.35	2.05E-02
11	*PTGFR*	prostaglandin F receptor	1.65	1.95	0.91	2.12E-02

Top 20 differentially expressed transcripts, as determined by adjusted p-value, for (A) 1-week surgical samples vs. controls, (B) 4-week surgical samples vs. controls, and (C) 52-week surgical samples vs. controls. Transcripts are ordered by adjusted p-value of REPAIR vs. controls. Only 11 transcripts are shown at 52 weeks because only 11 transcripts were shared among all surgical samples at that timepoint.

### Differential gene expression analysis by timepoint

**Fig 2:**
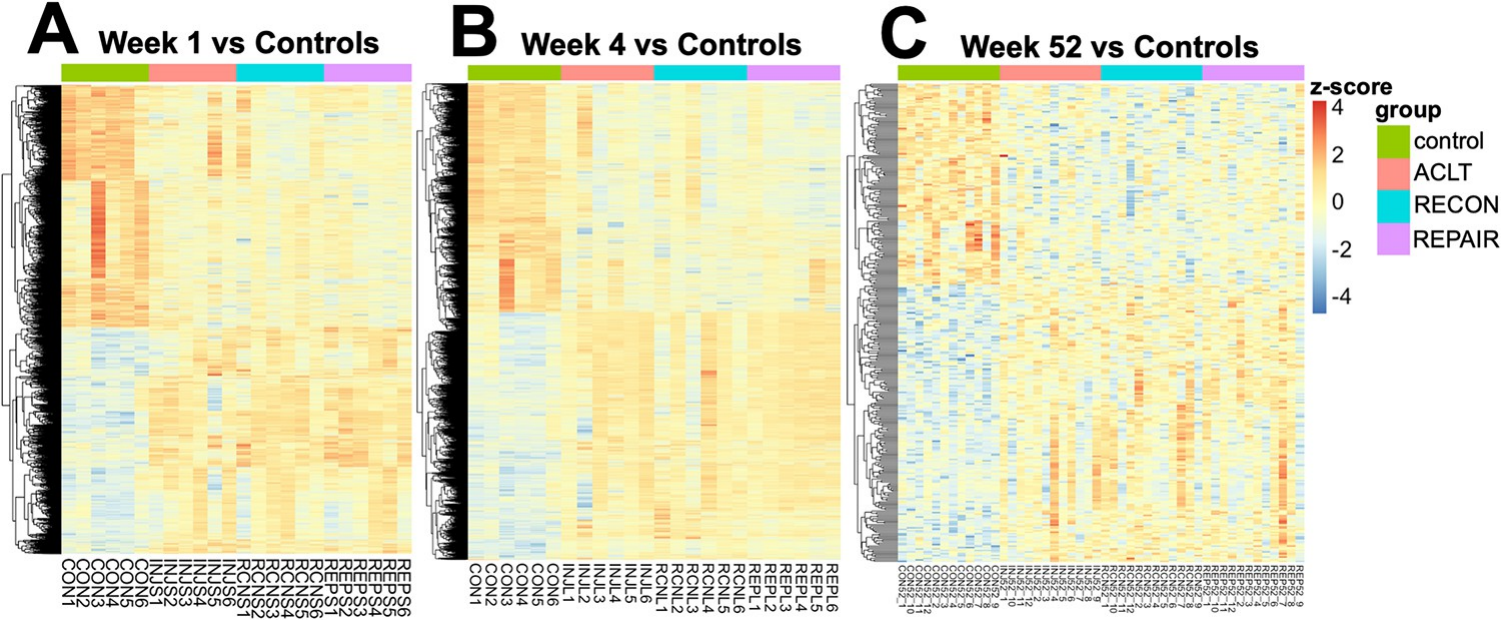
Heatmaps of significantly differentially expressed genes. Heatmaps of significantly differentially expressed genes for pooled timepoint ((A) 1-week, (B) 4-weeks, (C) 52-week) comparisons to controls. The red-blue color spectrum indicates the z-score for each gene. Surgical groups are labeled above columns, and subjects IDs are listed below columns.

There were significant differences in gene expression between controls and pooled surgical subjects at all timepoints (Table 5; exact p-values shown in Table 2 in S5 Appendix). At 1 week following surgical intervention, pooled surgical subjects featured 4206 differentially expressed transcripts compared to the no-surgery control group (Fig 2A). At 4 weeks, pooled surgical groups featured 5539 differentially expressed transcripts compared to the non-surgical controls (Fig 2B). And at 52 weeks, pooled surgical groups featured 314 differentially expressed transcripts compared to intact controls (Fig 2C). Across all timepoint comparisons, five genes were consistently differentially expressed: *MMP1*, *POSTN*, *IGF1*, *PTGFR*, and *HK1* (Table 6).

**Table 5:** The number of differentially expressed transcripts for each timepoint.

	1-Week			4-Week			52-Week
**Controls**	4206*			5539*			314*
**1-Week**				2776*			
	***Shared transcripts across all pooled comparisons to controls (top 100)***						
	98 (5)						
	***Unique transcripts***						
	30			16			81

The number of differentially expressed transcripts for each timepoint (p ≤ 0.05 represented by *). Shared transcripts among timepoints are shown with values in parentheses indicating how many transcripts are shared when the transcript list of each timepoint is narrowed to its top 100 most statistically significant transcripts. Unique transcripts (i.e., present in the top 100 most significant transcripts for a given comparison and absent from the top 500 most significant transcripts for similar comparisons) are also shown beneath timepoints.

**Table 6:** Differentially expressed transcripts shared among the top 100 most significant transcripts.

	*Gene Symbol*	*Description*	*1W Pooled/CON L2FC*	*4W Pooled/CON L2FC*	*52W Pooled/CON L2FC*	*4W Pooled/CON Adjusted P-value*
1	*C1QTNF3*	C1q and TNF related 3	3.23	3.64		6.15E-20
2	*COL6A3*	collagen type VI alpha 3 chain	2.10	2.23		6.15E-20
3	*IFNAR2*	interferon alpha and beta receptor subunit 2	1.57	1.63		6.15E-20
4	*PTGFRN*	prostaglandin F2 receptor inhibitor	1.22	1.52		4.22E-19
5	*MMP1*	matrix metallopeptidase 1	9.23	7.67	1.43	2.54E-18
6	*POSTN*	Periostin	5.75	6.34	1.53	3.55E-18
7	*IGF1*	insulin like growth factor 1	4.23	4.78	1.42	7.94E-18
8	*PTGFR*	prostaglandin F receptor	4.82	5.71	1.92	7.94E-18
9	*MEDAG*	mesenteric estrogen dependent adiposis	2.62	2.88		6.45E-16
10	*SNAP91*	synaptosome associated protein 91	-2.80	-2.16		1.61E-15
11	*BEST1*	bestrophin 1	-3.02	-3.16		3.10E-14
12	*RGS16*	regulator of G protein signaling 16	2.84	2.89		3.23E-14
13	*NCAPH*	non-SMC 13pregulate I complex subunit H	2.94	2.10		6.67E-13
14	*B4GALT1*	beta-1,4-galactosyltransferase 1	1.60	1.53		7.02E-13
15	*ABCB9*	ATP binding cassette subfamily B member 9	-2.34	-2.34		9.22E-13
16	*CKS2*	CDC28 protein kinase regulatory subunit 2	2.64	1.72		2.22E-12
17	*KIF20A*	kinesin family member 20A	3.74	2.57		1.06E-10
18	*SZRD1*	SUZ RNA binding domain containing 1	0.78	0.68		1.47E-10
19	*TK1*	thymidine kinase 1	3.68	2.61		1.54E-10
20	*CKAP2*	cytoskeleton associated protein 2	4.07	2.68		2.02E-10
21	*TPX2*	TPX2 microtubule nucleation factor	3.05	1.80		3.41E-10
22	*PIK3R3*	phosphoinositide-3-kinase regulatory subunit 3	2.60	1.97		3.90E-10
23	*STMN1*	stathmin 1	2.93	1.67		4.76E-10
24	*RRM2*	ribonucleotide reductase regulatory subunit M2	3.36	1.95		7.73E-10
25	*HK1*	hexokinase 1	1.32	0.99	0.33	8.89E-10
26	*TYMS*	thymidylate synthetase	3.27	1.86		1.07E-09
27	*SHCBP1*	SHC binding and spindle associated 1	4.08	2.74		1.68E-09
28	*STEAP3*	STEAP3 metalloreductase	2.63	2.11		2.21E-09

The 28 differentially expressed transcripts shared among the top 100 most significant transcripts from pooled 1-Week vs. controls and pooled 4-Week vs. controls, and the 5 transcripts which are shared with the top 125 most significant transcripts from pooled 52-Week vs. controls.

### Functional pathway analyses 1 week after surgery

**Fig 3:**
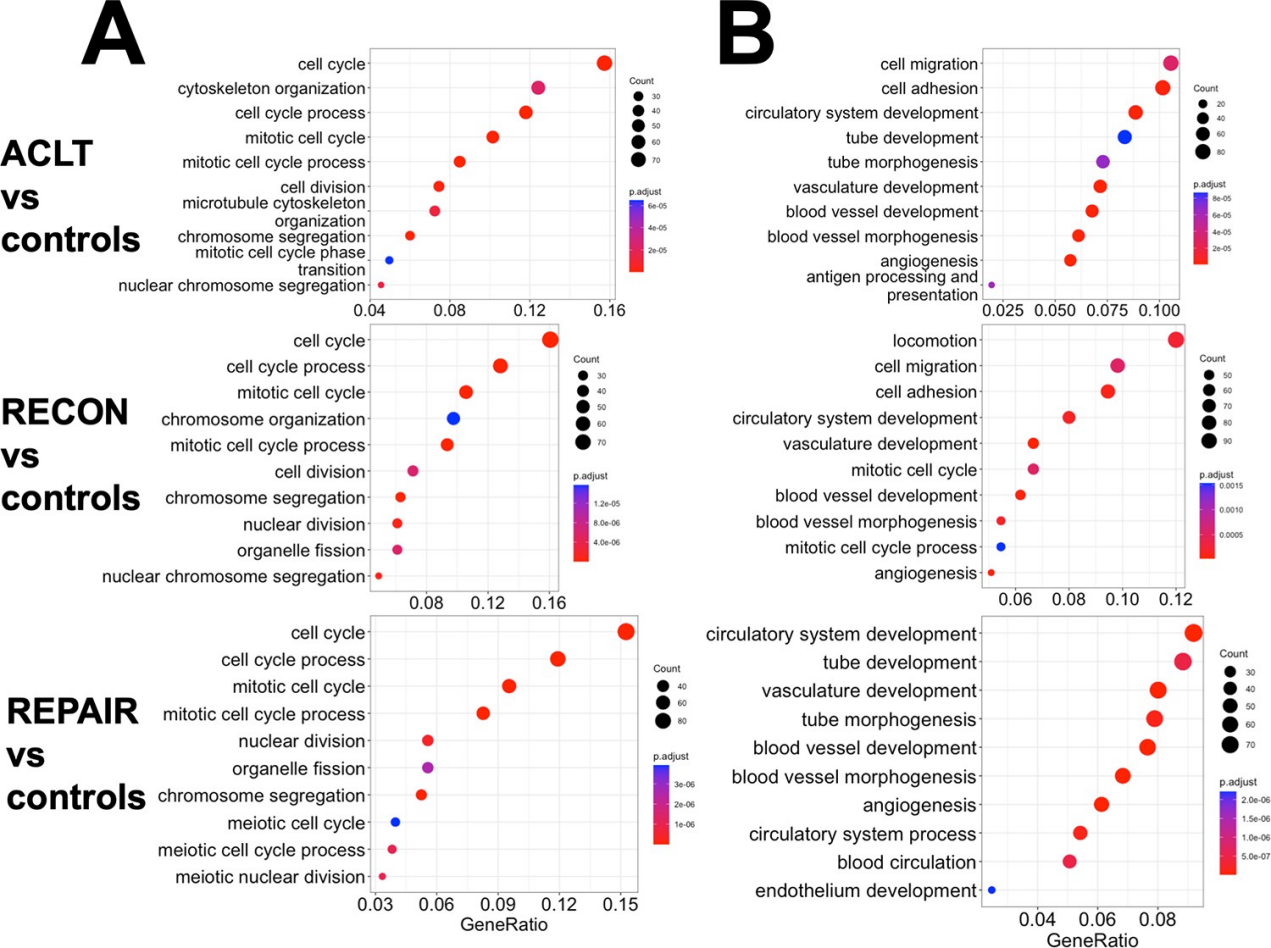
Top 10 upregulated biological process GO terms. The top 10 upregulated biological process GO terms based on overrepresentation analysis for (A) 1-week and (B) 4-week ACLT, RECON, and REPAIR compared to controls arranged by gene ratio (i.e., the number of genes related to the GO term divided by the total number of significant genes in the comparison). The number of genes represented by a term is indicated by the size of the circle, and the color represents the adjusted p value.

**Fig 4:**
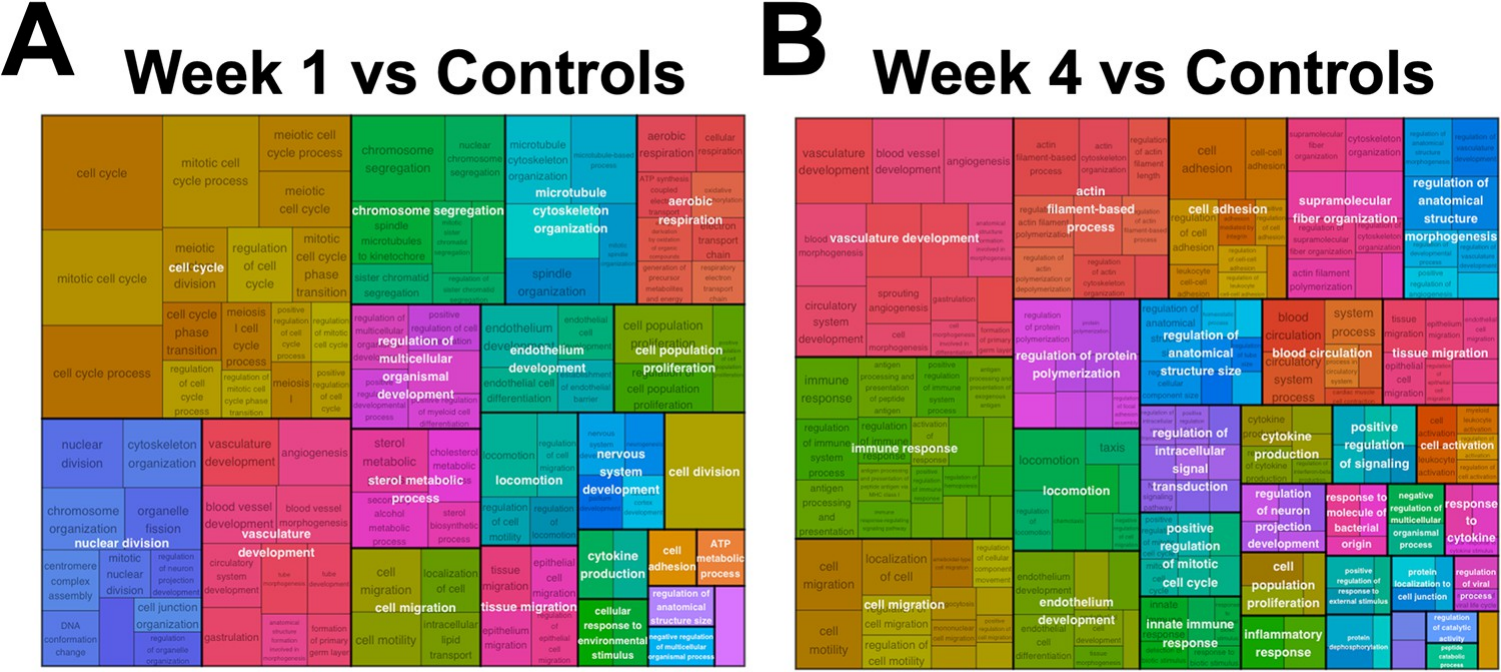
Treemap plots of upregulated biological process GO terms. Treemap plots of upregulated biological process GO terms for (A) pooled 1-week vs. controls and (B) pooled 4-week vs. controls. Terms are grouped and colored according to unifying parent terms, and the amount of space a term occupies is proportional to gene set size and hypergeometric testing of overrepresented genes.

At 1 week following surgery, pathways related to cell proliferation were the most enriched in the surgical groups compared to controls. This finding was supported by overrepresentation and upregulation of genes associated with the biological process *cell cycle* when surgical groups were compared to controls (Fig 3A). There was no overrepresentation of downregulated biological process GO terms for individual surgical group comparisons to controls. A treemap of biological process GO terms for pooled 1-week vs. controls was generated (Fig 4A). *Cell cycle* and *nuclear division* were the most notable parent terms, followed by *vasculature development* and terms related to the intranuclear mitotic arrangement and cellular movement.

**Fig 5:**
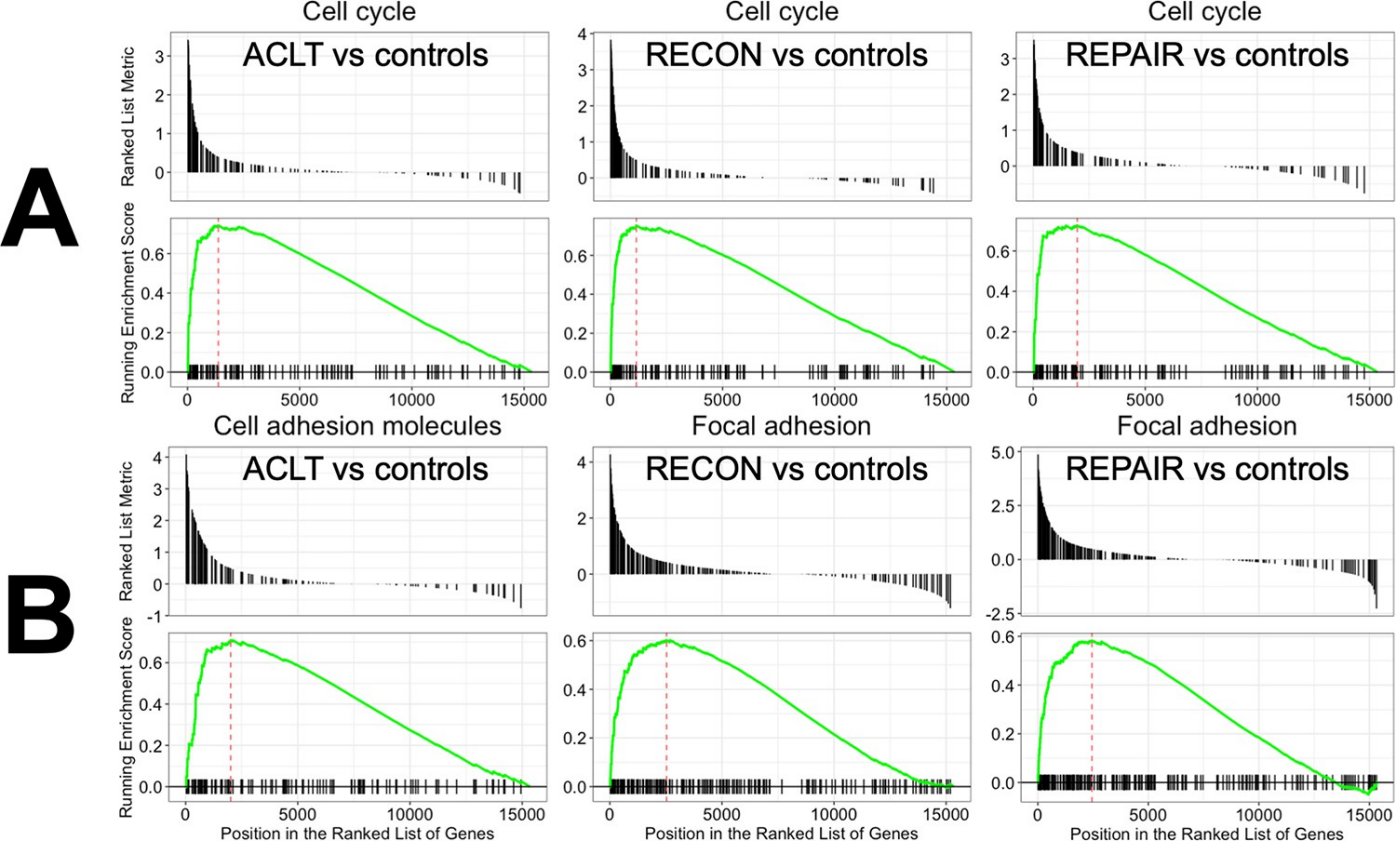
GSEA enrichment plots. GSEA enrichment plots for (A) 1-week ACLT, RECON, and REPAIR compared to controls and (B) 4-week ACLT, RECON, and REPAIR compared to controls. The upper portion of each plot has lines which represent the log2 fold changes for each gene considered in the KEGG pathway. The lower portion of each plot shows the running total of these log2 fold changes, with a positive deflection representing upregulation in the first group listed in the comparison relative to the second group listed (i.e., ACLT vs. controls—first vs. second).

GSEA demonstrated that the most significantly enriched pathway was *cell cycle* when all three surgical groups were individually compared to controls (Fig 5A). Complete lists of the KEGG terms with significant GSEA results for these comparisons and an SPIA plot and table can be found in S4 Appendix in the data availability repository. Most pathways were involved in *cell cycle*, *ECM-receptor interaction*, and *focal adhesion*.

### Functional pathway analyses 4 weeks after surgery

At 4 weeks after surgery, angiogenesis and cell adhesion pathways were the most enriched in the surgical groups compared to controls. This finding was supported by the overrepresentation and upregulation of genes related to the biological processes of *angiogenesis*, *cell migration*, and *cell adhesion* when compared to controls (Fig 3B). A treemap of biological process GO terms for pooled 4-week vs. controls revealed *vasculature development*, *immune response*, *cell migration*, *actin filament-based process*, and *cell adhesion* were the most represented parent terms (Fig 4B).

GSEA at 4 weeks demonstrated all three surgical groups featured *cell adhesion* or *focal adhesion* among their top 10 most significant terms as determined by adjusted p-value (Fig 5B). There was also upregulation of pathways related to inflammation and immune activation (e.g., *Staphylococcus aureus infection*). When individually assessed with SPIA, all 4-week surgical groups vs. controls featured upregulation of the terms *ECM-receptor interaction*, *focal adhesion*, *PI3K-Akt signaling*, *Calcium signaling pathway*, and complement-containing pathways. Concordantly, SPIA for the pooled 4-week vs. controls comparison featured upregulation of these terms within the top 10 most significantly represented pathways (S4 Appendix in the data availability repository).

REPAIR vs. RECON at 4 weeks following surgery featured a significant number of differentially expressed genes (Table 3). The top 5 biological process GO terms were all related to more expression in RECON than REPAIR of SMAD phosphorylation/signaling. These terms were influenced by the RECON group’s upregulation of *INHBA*, *GDF5*, *TGFB1*, and *BMP2*. GSEA featured increased expression in RECON relative to REPAIR of *TGFβ-*related terms, including *cytokine-cytokine receptor interaction*, *rheumatoid arthritis*, and *TGF-beta signaling pathway* (S4 Appendix in the data availability repository). Similarly, downregulation of TGFβ*-*related genes was involved in every SPIA-featured pathway: *cytokine-cytokine receptor interaction*, *Hippo signaling pathway*, *TGF-beta signaling pathway*, and *osteoclast differentiation* (GSEA plots available in S4 Appendix in the data availability repository).

Pooled 4-week surgical subjects were directly compared to pooled 1-week surgical subjects. Similar to above, *cell adhesion*, *positive regulation of immune system process*, and *vasculature development* were among the top upregulated biological process GO terms (S3 Appendix in the data availability repository).

### Functional pathway analyses 52 weeks after surgery

**Fig 6:**
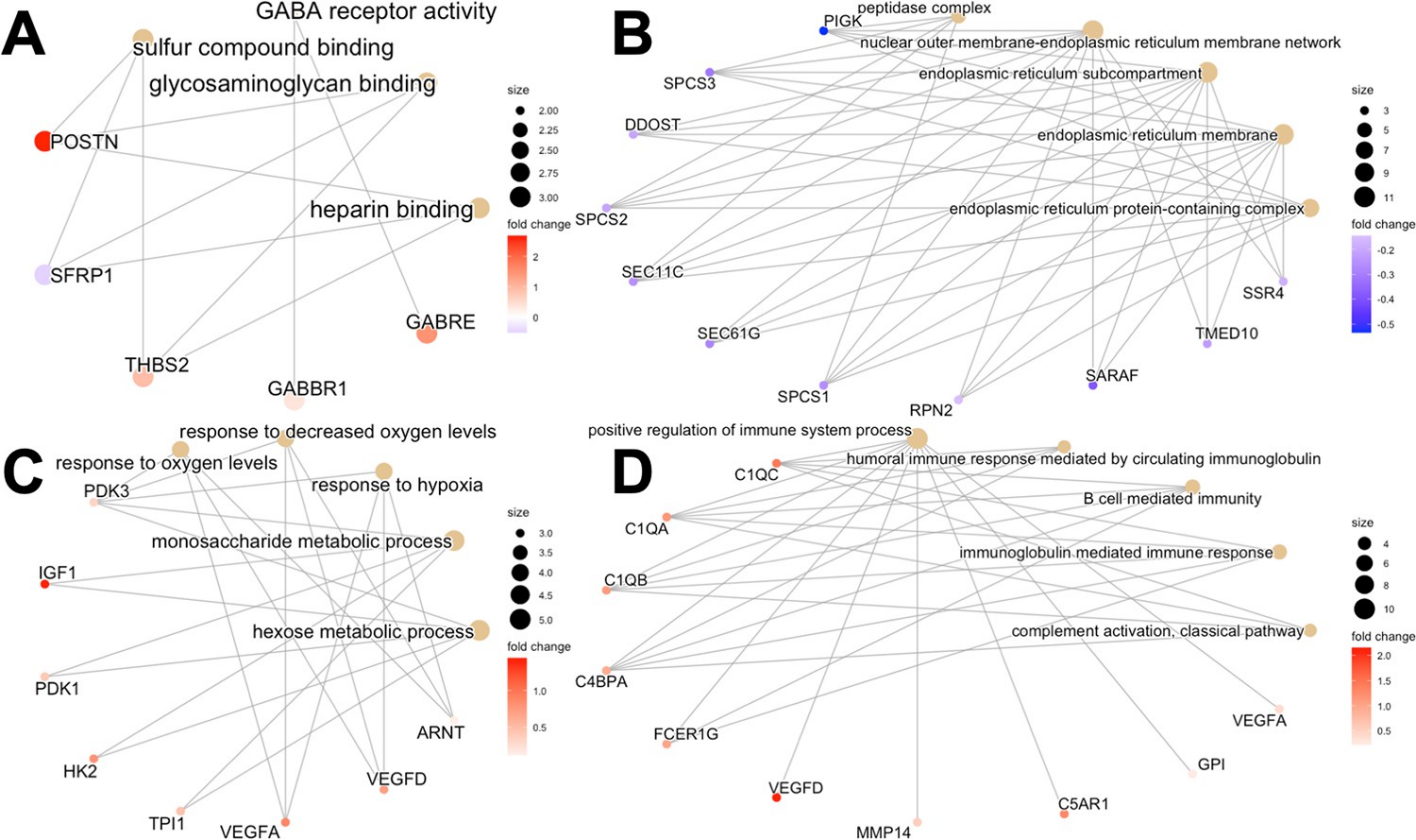
Category netplots. Category netplots for (A) 52-week ACLT vs. controls (molecular function GO terms), (B) RECON vs. controls (downregulated cellular component GO terms), (C) REPAIR vs. controls (upregulated biological process GO terms), and (D) pooled 52-Week vs. controls (upregulated biological process GO terms) based on overrepresentation analysis. GO term size is proportional to how many genes contribute to it, and fold change of expression is color coded.

At 52 weeks following surgery, surgical groups varied in their overrepresentation of GO terms across all three surgical groups compared to controls. ACLT vs. controls featured an overrepresentation of molecular function GO terms *heparin binding*, *glycosaminoglycan binding*, and *sulfur compound binding*—driven by *POSTN*, *SFRP1*, and *THBS2* without clear directionality of expression (Fig 6A). There was also overrepresentation and mild upregulation of genes related to *GABA receptor activity* in ACLT samples relative to controls (Fig 6A). RECON vs. controls (and ACLT vs. controls) featured an overrepresentation of downregulated genes related to endoplasmic reticulum membrane regulation (Fig 6B). REPAIR vs. controls featured upregulation of biological process GO terms *hexose metabolic process*, *monosaccharide metabolic process*, *response to hypoxia*, and *response to decreased oxygen levels* (Fig 6C). Pooled 52-week vs. controls featured molecular function GO terms with mixed expression (e.g., *glycosaminoglycan binding*), cellular component GO terms with downregulation (e.g., *endoplasmic reticulum membrane*), and biological process GO terms with increased expression (e.g., *positive regulation of immune system process*) (Fig 6D). REPAIR vs. RECON at 52 weeks featured upregulated molecular function GO terms *protein kinase binding* and *kinase binding* dependent upon the genes *NR4A3*, *MAPK6*, and *SOX9*.

GSEA at 52 weeks revealed that all three surgical groups (when compared to controls) featured upregulation of *calcium signaling pathway* among their top 10 most significant terms as determined by adjusted p-value. This pathway depends upon growth factor signaling, and each comparison featured several significantly upregulated growth factor genes. ACLT vs. controls featured upregulated *neuroactive ligand-receptor interaction* and *GABAergic synapse*. RECON vs. controls featured upregulation of *pathways in cancer* and *PI3K-Akt*, which were partly driven by growth factor signaling and ECM components of focal adhesion (S4 Appendix in the data availability repository). *Amoebiasis* was also upregulated, with contributions from laminin- and collagen-related genes. REPAIR vs. controls featured upregulation of the *HIF-1 signaling pathway*. This finding was driven by upregulation of HIF-, HK-, PDK-, VEGF-, and growth factor-related genes. Like ACLT vs. controls, *neuroactive ligand-receptor interaction*, *GABAergic synapse*, and *pathways in cancer* were upregulated for this comparison. Concordantly, SPIA for the pooled 52-week vs. controls featured upregulation of *calcium signaling pathway*, *focal adhesion*, *PI3K-Akt signaling pathway*, and *complement and coagulation* cascades (S4 Appendix in the data availability repository).

## Discussion

The current study involved 78 porcine subjects and produced 90 transcriptomes across timepoints that extended up to a year. Differential gene expression analysis showed how transcriptomic differences between PTOA cartilage and healthy cartilage evolve as the disease progresses. It also displayed on a genetic level how different treatments modulate the course of PTOA following ACL transection. Specific genes (e.g., *MMP1*, *POSTN*, *IGF1*, *PTGFR*, *HK1*) were identified as being upregulated in surgical subjects across all timepoints. Functional pathway analysis of surgical groups compared to healthy cartilage revealed overarching patterns of cellular proliferation at 1 week, angiogenesis, ECM interaction, focal adhesion, and migration at 4 weeks, and calcium signaling, immune system activation, GABA signaling, and HIF-1 signaling at 52 weeks.

Overall, the number of differentially expressed genes increased (i.e., from 1 week to 4 weeks) and then substantially decreased (i.e., from 1 and 4 weeks to 52 weeks) when surgical groups were compared to controls. This trend suggests that, following injury, chondrocytes have a pronounced, reactive transcription phase that builds over the first few weeks of injury and then quiesces over a year. *MMP1*, *POSTN*, *IGF1*, *PTGFR*, and *HK1* were still shared and upregulated among all pooled timepoint comparisons to controls, which suggests an enduring, typical response that all joints had following trauma.

*MMP1* has repeatedly been shown to be upregulated in articular cartilage following surgical induction of PTOA in animal models—with expression positively correlated to time since injury and degree of cartilage damage for up to 12 weeks after surgery [13, 36, 37]. The current study demonstrates this continues through 52 weeks after surgery. Although much of the documentation of MMP-1 in the osteoarthritis literature is related to its role in cleaving type II collagen in articular cartilage, this matrix-degrading protease also has pivotal roles in modulating inflammatory cell recruitment, processing inflammatory cytokines, and activating VEGF to dilate pre-existing capillaries [38, 39]. The continued expression of *MMP1* in injured cartilage suggests that these molecular pathways may remain active long after the initial insult.

Several studies have implicated periostin (encoded by *POSTN*) in the biology of cartilage degeneration [40]. Murine PTOA models have shown peak *POSTN* expression at 2–6 weeks [41] and diminished microscopic cartilage damage in *POSTN* knockouts [42]. In the current study, ACL transection in the porcine model featured consistent upregulation of *POSTN* at 1, 4, and 52 weeks relative to uninjured cartilage. This, along with evidence that *POSTN* expression is elevated in end-stage disease in human cartilage [43], suggests that chondrocyte periostin production may rise significantly in the early stages of cartilage compromise and continue at a lower (but still elevated) level in the subsequent months to years.

IGF-1 is known to have chondroprotective effects, such as stimulating neocartilage formation, promoting cell survival, and enhancing the integrity of ECM [44, 45]. A recent posttraumatic murine model has shown that subpopulations of resident knee joint macrophages constitute a significant source of this *IGF1* expression, and chondrocytes (expectedly) have high expression of *IGF1R* in the 3–7 days following compressive ACL rupture [46]. In the current porcine study, expression of *IGF1* was elevated at 7 days, and it remained elevated at 4 and 52 weeks following ACL transection—suggesting that cartilage may work to maintain a chondroprotective effort even a year after injury. Ongoing endeavors are to create IGF-1 delivery systems that increase this promising growth factor’s duration and cartilage penetration [47, 48]. The present findings suggest that exogenous agents could longitudinally work in parallel with native cellular efforts.

There is less documentation of *PTGFR* expression as it relates to the development of posttraumatic osteoarthritis [17]. There is evidence that the PGF_2α_ receptor (encoded by *PTGFR*) is upregulated during chondrocyte differentiation and that it promotes ECM production (e.g., collagen type II and aggrecan) [49, 50]. Activation of this receptor has also been noted to hypertrophy chondrocytes, which mineralize the surrounding matrix and release VEGF [49]. In vitro testing has shown that chondrocyte PGF_2α_ expression can be induced by shear stress, and administration of a COX-2 inhibitor can prevent this response—a finding that is concordant with the relief that NSAIDs often provide osteoarthritis patients, who have been found to have elevated PGF_2α_ in serum and synovial fluid [51, 52]. Aside from being a potential source of pain, PGF_2α_ may also play a substantial role in profibrotic changes and joint stiffening as osteoarthritis progresses [53]. Considering this evidence, yearlong *PTGFR* upregulation, as seen in the current study, might indicate an inflammatory mechanism induced by shear stress that promotes cartilage regeneration but eventually leads to cartilage mineralization. Further evidence that this mechanism becomes unbalanced over time is the significant expression of *PTGFRN* at 1 and 4 weeks but not at 52 weeks (i.e., *PTGFR* expression may be balanced by *PTGFRN* expression at earlier timepoints). Given its dynamic, opposed, and then unopposed expression in posttraumatic osteoarthritis, *PTGFR* is an understudied and promising target for future PTOA research.

Hexokinase 2 (HK2) has been a focus of osteoarthritis research in recent years. Still, another hexokinase isoform, HK1, has been less studied. Both isoforms have been found in cultured chondrocytes from osteoarthritis patients [54]. *HK2* expression has been identified in fibroblast-like synoviocytes from osteoarthritis knees. It can be upregulated by TNF and hypoxia, which can increase the production of inflammatory cytokines if *HK2* is overexpressed [55]. Both HK1 and HK2 serve as the first rate-limiting enzyme in glycolysis, implicating their involvement in metabolic derangements in osteoarthritis chondrocytes. There is evidence that chondrocytes undergo a metabolic shift from a state of regulatory rest to a state of high metabolic activity to meet the homeostatic needs required for cell survival [56]. Usually, this occurs in conditions of low oxygen tension, but it has been noted to occur in normoxic contexts such as cancer (i.e., Warburg effect) and degenerative/inflammatory diseases where HIF-1α plays a mediating role [56]. In end-stage osteoarthritis, chondrocyte hypoxia has been linked to chondrocyte apoptosis, cell senescence, dysregulated ECM, and increased abundance of ROS [57]. The dysregulation of ECM likely occurs because glucose provides fuel for cartilage development and is a precursor to glycosaminoglycans which are vital to ECM construction [58]. This intertwining of glucose processing and its HIF-1α-related regulation suggests that HK1 may be a principal agent in mediating changes in chondrocyte metabolism and its impacts on ECM degeneration. The current study found that *HK1* was consistently upregulated at 1, 4, and 52 weeks relative to controls—evidence that challenges inconsistent findings in the literature, which have found *HK1* levels in end-stage cartilage to be higher or no different than in healthy cartilage [55, 59].

Aside from identifying genes that are concordantly significant at 1, 4, and 52 weeks (Table 6), the current study also independently characterized the transcriptome of the PTOA porcine model at 52 weeks (Table 4C and Table 3 in S5 Appendix). This is a mid-range timepoint, which to the best of our knowledge, has yet to be explored in the literature—mainly due to logistical, physical, and financial hurdles that must be overcome to maintain a large animal model for over a year. There were 11 differentially expressed genes that were concordantly regulated across the top 100 gene lists of all 52-week surgical groups compared to controls (Table 4C). Four of these genes (*A4GALT*, *EFS*, *NPTXR*, *ABCA3*) have virtually no association with osteoarthritis or cartilage in the literature. They, therefore, represent new targets that have only been identified one year into PTOA development. Alternatively, six of the top concordant genes (*HTR7*, *TRAK1*, *LAMA4*, *HS6ST1*, *PDE4A*, *GPNMB*) at 52 weeks have been documented in prior osteoarthritis literature. Still, their identification in the current study implicates them at a timepoint in disease pathogenesis that was previously unknown [60–69].

When 52-week cartilage samples were stratified into less and more damaged groups, only 3 transcripts were differentially abundant. There are several potential reasons for the minor transcriptional difference between damage groups. Surgical groups may be mediated by different genetic signals, and pooling these groups may mix/obscure these different signals. For instance, REPAIR subjects with more damage may develop that damage in a way that is distinct from how ACLT subjects developed the same damage. Grouping these subjects may muddle the discernment of their individual mechanisms. However, if this were the sole explanation, one would expect a significant expression difference between REPAIR and ACLT, which there was not. Another possible reason is that cartilage differences between surgical groups and controls are substantial enough to produce a detectable transcriptomic signature, but cartilage differences among surgical groups are not significant enough to produce such a detectable signature—at least not at this timepoint. And lastly, the poor transcriptomic distinction between more and less damaged cartilage may indicate that we are dealing with a disease that has less transcriptional (and more mechano-physical) influence at 52 weeks than was previously thought.

Functional pathway analysis at 1 week was dominated by evidence of cell cycle upregulation. This suggests that the earliest phases of the chondrocyte reaction to joint injury are related to compensatory cellular proliferation, which has been associated with reduced chondrocyte apoptosis and increased secretion of cartilage matrix [70]. The unequivocal strength of this early proliferative signal in the cartilage of the current study stands in stark contrast to the varied role of the cell cycle pathway in the osteoarthritis literature, which has depicted the cell cycle as either not being enriched at 1 week in a murine PTOA model or possibly downregulated in a 1- to 9-week equine model [10, 16]. However, the latter evaluated synovial fluid for microRNA expression and did not characterize the cartilage or its cellular contents. An additional murine *POSTN* knockout, which protected against PTOA, showed downregulation of cell cycle pathways at 8 weeks after injury [42]. Human studies in end-stage disease have also found cell cycle pathways to be enriched, and their constituent genes were downregulated or had mixed expression directionality [71, 72]. Single-cell sequencing of end-stage human osteoarthritis cartilage further revealed a subpopulation of cartilage progenitor cells (CPCs) that has an enriched upregulation of cell cycle, chromosomal organization, and DNA replication pathways, but most cell types did not feature this enrichment [73]. The current and previous studies suggest that cellular proliferation may be a very early (i.e., ~1 week) phenomenon following the induction of osteoarthritis. An overwhelming compensatory surge in cellular division may occur following an injury that quiesces and then downregulates in the following weeks. And as the end-stage human studies have shown, the cartilage cellular profile eventually settles into a state where a select few chondrocytes remain reproductively active, but the majority do not.

Vasculature development, focal adhesion, and ECM-receptor interaction were upregulated at 1 week, and, along with cellular migration and immune response, became the predominantly upregulated pathways at 4 weeks. This is concordant with previous animal models, which have shown mild cartilage vascular development 7 days after disease induction and more prominent vascularization by 14 and 28 days [74]. Mechanistically, this angiogenesis is enabled by osteoclasts, which pave channels from the subchondral bone through the tidemark into normally avascular non-calcified cartilage [75]. These channels result in subchondral bone marrow being supplanted by *VEGF*-expressing fibrovascular tissue [76]. This process has been documented in rodents from 2 to 6 weeks following destabilization of the medial meniscus and in various stages of human disease when assessed in non-standardized post-mortem patients [77, 78]. Thus, these processes are not restricted to early- or late-stage disease. Along with this vascularization of non-calcified cartilage come exclusively perivascular sensory and sympathetic nerve fibers, which are believed to be a source of pain as the disease progresses [79]. On the other hand, upregulation of focal adhesion and ECM interaction pathways have been found in early- and end-stage osteoarthritis cartilage and—in the latter—may indicate collagen fibrosis and conversion of chondrocytes to fibroblasts [80, 81]. Additionally, end-stage enrichment of these pathways may indicate monocyte adhesion and migration into the synovium or differentiation (e.g., monocytes into macrophages or macrophages into osteoclasts) [82]. Therefore, enrichment of angiogenesis, adhesion, ECM, and cellular migration pathways at 4 weeks suggests a transition from chondrocyte replication at 1 week. Instead, there is a focus on the creation of osteoclasts that drive neovascularization, which lays the foundational infrastructure for future nerve pain, changing the cellular profile to one of fibrosis creation, and (through upregulation of MAPK signaling) stimulating the release of MMPs [83, 84]. Additionally, the upregulation of adhesion pathways in the current study may indicate some infiltration of immune cells through vascularized non-calcified cartilage networks. However, this infiltration has only been previously documented in synovium [76].

At 4 weeks following surgery, there was distinct enrichment of TGFβ-related pathways in RECON relative to REPAIR. Several studies have shown increased TGFβ signaling in cartilage associated with early-stage synthetic activity and chondroprotective mechanisms [85, 86]. This TGFβ upregulation is believed to be a temporary anabolic impulse that eventually loses out to catabolic pathways once TGFβ expression declines [86]. The entire mechanism of TGFβ-related signaling remains unresolved, but it does involve the interplay of SMAD family expression and transcription factors downstream of MAPK signaling [86]. There is evidence that SMAD2/3, like the TGFβ family constituents they interact with, decline in chondrocytes as osteoarthritis progresses while SMAD1/5/8 increase [87, 88]. The former stymies chondrocyte differentiation while the latter promotes it [87–89]. Increased TGFβ signaling in RECON compared to REPAIR subjects might suggest increased anabolic activity and less chondrocyte differentiation in the RECON group—something that contradicts the cartilage damage differences seen between the groups at 1 year. Perhaps, up until 4 weeks, the REPAIR subjects have incurred less damage than the RECON subjects and thus have less upregulation of compensatory mechanisms. Additionally, *BMP2* expression is tied to TGFβ signaling, but there is conflicting evidence regarding its association with osteoarthritis progression [90, 91]. Similarly, *GDF5*, another member of the TGFβ family, has conflicting evidence; several studies relate *GDF5* upregulation to ECM production and collagen type II production following osteoarthritis induction while others show reduced or absent expression in late-stage disease or soon after DMM [92–94]. Reduced *GDF5* expression in late-stage disease or severely damaged cartilage may suggest that expression increases in early-stage disease but attenuates as degeneration progresses. In the current study, *BMP2* and *GDF5* were upregulated at 4 weeks in RECON compared to REPAIR subjects—associating these genes with worse cartilage outcomes following injury.

At 52 weeks following surgery, all surgical groups featured upregulation of calcium signaling and upregulation of growth factor constituent genes. This activation of calcium pathways may represent mechanosignaling or voltage-gated calcium regulation. The former requires non-motile cilia that protrude from chondrocytes and increase in length and number as osteoarthritis worsens [95, 96]. Mechanical perturbation of these cilia, modulated by growth factor signaling (i.e., TGFβ and BMPs), induces calcium influx into chondrocytes and plays a central role in downstream MAPK and β-catenin signaling [97]. Voltage-gated calcium signaling, on the other hand, has been much less studied in osteoarthritis, but there is evidence that administering antagonists to these channels could stymie osteoarthritis symptoms and the development of cartilage lesions following injury [14, 98]. Regardless of the predominant mechanism, the current study has shown that expression of genes related to calcium signaling is the most conserved genetic signal across all surgically induced PTOA subjects at 52 weeks. Additionally, the upregulation of γ-aminobutyric acid (GABA) receptor expression was prominently featured in pathway analysis for ACLT and REPAIR subjects. This finding, along with recent evidence that blocking GABA breakdown prevents cartilage degeneration after injury, suggests that GABAergic pathways remain relevant to osteoarthritis even one year after injury [99]. GABA signaling may also impact pain transmission as perivascular innervation forms in the non-calcified cartilage following injury. Lastly, there was upregulation of hypoxia pathways for REPAIR subjects at 52 weeks. There is an established connection between chondrocyte hypoxia and deleterious cartilage outcomes, and HIF-1α has been shown to be upregulated as a compensatory force for chondrocyte differentiation and survival [56, 100]. Nevertheless, some analyses of end-stage human cartilage have shown conflicting relationships between the expression of HIF-1-related genes and the degree of cartilage damage [8, 100]. The current study helps resolve this conflict by associating upregulation of the HIF-1 pathway in the surgical group with the best cartilage outcomes—strengthening the notion that HIF-1 has chondroprotective effects. When all 52-week surgical subjects were pooled together and compared to controls, there was a notable upregulation of humoral- and complement-mediated immune pathways. Immunoglobulins and immune complexes against cartilage, along with complement activation, have previously been identified in the cartilage of end-stage osteoarthritis patients [101, 102]. Complement likely comes from the surrounding synovial fluid (rather than from leakage from blood) [103]. The current study reaffirms the presence of these immune system compounds and implicates them at 52 weeks following joint trauma.

The current study is not without its limitations. One limitation is that the control group for the 1- and 4-week subjects had no prior surgery on either knee while the 52-week control group was composed of contralateral knees from subjects that had undergone surgery. This was done due to the prohibitive cost of humanely keeping animals alive through the 52-week timepoint only as controls. This decision was also supported by evidence that subchondral bone and cartilage thickness do not differ between surgery-naïve controls and contralateral knees of humans who have suffered ACL tears [104, 105]. Similar evidence of healthy contralateral knees following ACL injury has also been seen in quadrupeds, even 54 months after ACL transection [106]. Consistent with that prior quadruped study, we did not see significant differences in control groups macroscopically (Table 2), suggesting that at least macroscopic damage at this timepoint after ACL surgery was not different in the two control groups. While the 1-, 4-, and 52-week surgical and control samples all underwent the same RNA-Seq processing (which has substantially changed since prior reporting in 2018 of the short-term samples with an updated Sus scrofa genome, mapping algorithms, and gene expression analysis software) and utilized the same animal model, interventional procedures, and type of data (e.g., fastq files for RNA-Seq analysis), we recognize there may be differences in the gene expression in the two control groups that may confound results when normalized to controls. However, we felt this risk was mitigated as qualitative—not quantitative—transcriptomic comparisons were made between 1- and 4-week findings and 52-week findings. That is, log fold changes were not calculated between these studies, and differential gene expression analysis was not performed between short-term and long-term tissues. Rather, patterns were noted in the ways genes/pathways were differentially regulated within respective timepoints. This respects the boundary established by the different controls as well as the fact that short- and long-term samples were sequenced separately. 52-week samples were sequenced separately from 1- and 4-week samples because specimens came from studies that were performed at different times. Ideally, all samples would be sequenced together to allow for direct fold change comparisons to be made across all timepoints. Nevertheless—as mentioned above—sequencing samples separately still allows for qualitative comparisons to be made. Lastly, OARSI cartilage scoring guidelines were designed for goats and sheep but have been extended to pigs for this study.

The overwhelming majority of osteoarthritis basic science research uses samples from the end-stage disease, which has left a vacuum in the understanding of the early- and mid-stage [6–9]. The current study characterized the transcriptional profile of PTOA at 1, 4, and 52 weeks following injury—thereby creating a novel time course for understanding the trajectories of differential gene expression and molecular dynamics in the year following ACL disruption. This study revealed that transcriptomic differences between PTOA cartilage and healthy cartilage increase early and decrease as the disease progresses. It also showed on a genetic level how different treatments modulate the course of PTOA following ACL transection. Specific genes were identified as being upregulated in surgical subjects across all timepoints—reinforcing or, in the case of *PTGFR* and *HK1*, establishing their links to PTOA pathogenesis. At the 52-week timepoint, 4 genes (e.g., *A4GALT*, *EFS*, *NPTXR*, *ABCA3*) that were previously unassociated with PTOA were identified as being concordantly differentially expressed across all treatment groups when compared to controls. Functional pathway analysis of surgical groups compared to healthy cartilage also revealed overarching patterns of cellular proliferation at 1 week, angiogenesis, ECM interaction, focal adhesion, and migration at 4 weeks, as well as calcium signaling, immune system activation, GABA signaling, and HIF-1 signaling at 52 weeks.

## Supporting information

S1 Appendix Detailed animal procedures. (DOCX)

S2 Appendix Demographic data. (XLSX)

S3 Appendix MultiQC general statistics. (HTML)

S4 Appendix MultiQC report for short-term cartilage. (XLSX)

S5 Appendix Randomization testing and top 20 pooled vs. control transcripts. (DOCX)

S6 Appendix MultiQC report for long-term cartilage. (HTML)
