# Supplementary material for: Transcriptomic changes in porcine articular cartilage one year following disruption of the anterior cruciate ligament
Source: PLoS One. 2023 May 3;18(5):e0284777. doi: 10.1371/journal.pone.0284777 (PMC10156018; doi:10.1371/journal.pone.0284777)
Supplement: S5 Appendix — (DOCX) [file pone.0284777.s005.docx]

**Table 1**: Randomization testing p values for pairwise comparison of experimental groups.

|  | **1-Week** |  |  | | **4-Week** | | | **52-Week** | |  |  |
| --- | --- | --- | --- | --- | --- | --- | --- | --- | --- | --- | --- |
|  | **ACLT** | **RECON** | **REPAIR** | | **ACLT** | **RECON** | **REPAIR** | **ACLT** | | **RECON** | **REPAIR** |
| **Controls** | p=0.03 | p=0.005 | p=0.005 | | p=0.01 | p=0.005 | p=0.005 | p=0.01 | | p=0.01 | p < 0.001 |
| **ACLT** |  | p=0.19 | p=0.085 | |  | p=0.155 | p=0.28 |  | | p=0.285 | p=0.13 |
| **RECON** |  |  | p=1 |  | |  | p=0.05 |  | |  | p=0.04 |
|  |  |  |  | |  |  |  |  | **Cartilage damage ≥ 9** | | |
|  |  |  |  | |  |  |  | **Cartilage damage ≤ 8** p=0.125 | | | |

**Table 2**: Randomization testing p values for pooled timepoint comparisons.

|  | **1-Week** |  |  | **4-Week** | | **52-Week** |  |  |
| --- | --- | --- | --- | --- | --- | --- | --- | --- |
| **Controls** | p < 0.001 | |  | p < 0.001 |  | p=0.005 |  |  |
| **1-Week** |  |  |  | p < 0.001 |  |  |  |  |

**Table 3:** The top 20 differentially expressed transcripts of pooled 52-week subjects compared to controls as determined by adjusted p-value.

|  | **Gene Symbol** | **Description** | **52W Pooled/CON L2FC** | **52W**  **Pooled/CON Adjusted P-value** |
| --- | --- | --- | --- | --- |
| 1 | *LAMA4* | laminin subunit alpha 4 | 0.691 | 1.79E-06 |
| 2 | *HTR7* | 5-hydroxytryptamine receptor 7 | 1.230 | 2.92E-06 |
| 3 | *VSTM2A* | V-set and transmembrane domain containing 2A | -3.296 | 4.40E-06 |
| 4 | *TRAK1* | trafficking kinesin protein 1 | 0.455 | 5.53E-06 |
| 5 | *ABCA3* | ATP binding cassette subfamily A member 3 | 0.595 | 8.16E-06 |
| 6 | *S100A4* | S100 calcium binding protein A4 | 2.206 | 1.26E-05 |
| 7 | *CHRDL1* | chordin like 1 | 1.706 | 1.26E-05 |
| 8 | *PDE4A* | phosphodiesterase 4A | 0.444 | 1.81E-05 |
| 9 | *AQP5* | aquaporin 5 | -2.328 | 2.62E-05 |
| 10 | *GPNMB* | glycoprotein nmb | 2.619 | 2.62E-05 |
| 11 | *HS6ST1* | heparan sulfate 6-O-sulfotransferase 1 | 0.578 | 4.37E-05 |
| 12 | *A4GALT* | alpha 1,4-galactosyltransferase | 1.023 | 4.37E-05 |
| 13 | *CA9* | carbonic anhydrase 9 | 1.571 | 4.37E-05 |
| 14 | *RGP1* | RGP1 homolog, RAB6A GEF complex partner 1 | -1.379 | 4.63E-05 |
| 15 | *FGF7* | fibroblast growth factor 7 | 2.524 | 4.63E-05 |
| 16 | *GABBR1* | gamma-aminobutyric acid type B receptor subunit 1 | 0.310 | 7.39E-05 |
| 17 | *VEGFD* | vascular endothelial growth factor D | 2.153 | 8.82E-05 |
| 18 | *SCN4A* | sodium voltage-gated channel alpha subunit 4 | -2.683 | 8.82E-05 |
| 19 | *EFS* | embryonal Fyn-associated substrate | 0.421 | 9.67E-05 |
| 20 | *SCML4* | Scm polycomb group protein like 4 | 0.955 | 1.02E-04 |
